# High Periventricular T1 Relaxation Times Predict Gait Improvement After Spinal Tap in Patients with Idiopathic Normal Pressure Hydrocephalus

**DOI:** 10.1007/s00062-022-01155-0

**Published:** 2022-04-07

**Authors:** Ilko L. Maier, Marielle Heide, Sabine Hofer, Peter Dechent, Ingo Fiss, Christian von der Brelie, Veit Rohde, Jens Frahm, Mathias Bähr, Jan Liman

**Affiliations:** 1 Department of Neurology, University Medical Center Göttingen, Robert-Koch-Str. 40, 37075 Göttingen, Germany; 2 Biomedizinische NMR, Max-Planck-Institut für biophysikalische Chemie, Göttingen, Germany; 3 Institute for Cognitive Neurology, University Medical Center Göttingen, Göttingen, Germany; 4 Department of Neurosurgery, University Medical Center Göttingen, Göttingen, Germany

**Keywords:** Gait apraxia, CSF tap test, Communicating hydrocephalus, T1 mapping, T1 relaxometry

## Abstract

**Purpose:**

The diagnosis of idiopathic normal pressure hydrocephalus (iNPH) can be challenging. Aim of this study was to use a novel T1 mapping method to enrich the diagnostic work-up of patients with suspected iNPH.

**Methods:**

Using 3T magnetic resonance imaging (MRI) we prospectively evaluated rapid high-resolution T1 mapping at 0.5 mm resolution and 4 s acquisition time in 15 patients with suspected iNPH and 8 age-matched, healthy controls. T1 mapping in axial sections of the cerebrum, clinical and neuropsychological testing were performed prior to and after cerebrospinal fluid tap test (CSF-TT). T1 relaxation times were measured in 5 predefined periventricular regions.

**Results:**

All 15 patients with suspected iNPH showed gait impairment, 13 (86.6%) showed signs of cognitive impairment and 8 (53.3%) patients had urinary incontinence. Gait improvement was noted in 12 patients (80%) after CSF-TT. T1 relaxation times in all periventricular regions were elevated in patients with iNPH compared to controls with the most pronounced differences in the anterior (1006 ± 93 ms vs. 911 ± 77 ms; *p* = 0.023) and posterior horns (983 ± 103 ms vs. 893 ± 68 ms; *p* = 0.037) of the lateral ventricles. Montreal cognitive assessment (MoCA) scores at baseline were negatively correlated with T1 relaxation times (r < −0.5, *p* < 0.02). Higher T1 relaxation times were significantly correlated with an improvement of the 3‑m timed up and go test (r > 0.6 and *p* < 0.03) after CSF-TT.

**Conclusion:**

In iNPH-patients, periventricular T1 relaxation times are increased compared to age-matched controls and predict gait improvement after CSF-TT. T1 mapping might enrich iNPH work-up and might be useful to indicate permanent shunting.

**Supplementary Information:**

The online version of this article (10.1007/s00062-022-01155-0) contains supplementary material, which is available to authorized users.

## Introduction

Idiopathic normal pressure hydrocephalus (iNPH, also known as Hakim-Adams syndrome) is characterized by the neuropsychiatric symptom triad of gait disturbance, dementia and urinary incontinence [1]. Slowly progressive gait disturbances and falls are the most prominent clinical hallmark of the disease [2] and are often accompanied by progressive cognitive impairment and urinary incontinence. In contrast to symptomatic forms of secondary communicating hydrocephalus, e.g. after intraventricular hemorrhage or bacterial meningitis, patients with iNPH do not have a clear incidental event of the gradual and often insidiously progressive deterioration. The diagnosis of iNPH is based on clinical features and the history of evolving symptoms as well as on consistent imaging and spinal tap findings. In iNPH, cerebrospinal fluid (CSF) opening pressures typically are within a normal range and removal of high CSF volumes (known as the CSF-tap test (TT)) is associated with clinical improvement [3]. The improvement, especially of the gait disturbance, after CSF-TT further confirms the iNPH diagnosis and often leads to the indication of ventriculoperitoneal (VP) shunting, representing a highly effective treatment strategy in iNPH patients [3–6]; however, in these patients shunting itself bears possible complications like delirium, infections and overdrainage-associated subdural hematomas [7, 8]. Therefore, the indications for surgery must be based on clinical symptoms and imaging, improvement after spinal tap and the exclusion of a wide range of age-related differential diagnoses. In addition, selection criteria for VP shunting vary in the literature and also initial response to shunting does not necessarily translate into long-term response, which has been reported in the range between 29% and 80% of cases, possibly caused by false positive responses in patients with aqueduct stenosis, secondary NPH and placebo responses [8, 9].

In most institutions, either the CSF-TT or lumbar drain trial is used as confirmatory test and to indicate permanent shunting [3, 10]. Although these tests are widely accepted in clinical practice, they have no clearly defined sensitivity and specificity [11] and pretest probability of response is confounded by a high clinical variability and overlap of age-associated comorbidities affecting all three hallmarks of the disease [1]. These contributing comorbidities and differential diagnosis relevant in the diagnostic work-up of iNPH patients include cerebrovascular disease [12], neurodegenerative diseases like Alzheimer’s disease, musculoskeletal degeneration causing spinal canal stenosis, polyneuropathy and many others [13].

To overcome the problem of a solely clinical diagnosis of iNPH, various imaging hallmarks of the disease have been established. These hallmarks include disproportionate enlargement of the inner ventricles (also previously described as disproportionately enlarged subarachnoid space hydrocephalus, DESH) [14], increased Evans index (> 0.3) [15], a callosal angle between 40° and 90° [16] and periventricular white matter changes, accentuated around the anterior and posterior horn of the lateral ventricles. The latter is likely to be caused by transependymal egress of CSF, however, with a large overlap of microangiopathy being highly prevalent in this patient group [1, 17]. All these observations vary largely in their sensitivity and specificity and still have to be consistent with the also widely varying clinical presentation of iNPH patients. Nonetheless, the imaging characteristics of iNPH can also be found in the majority of differential diagnoses like Alzheimer’s disease or subcortical arteriosclerotic encephalopathy (deep periventricular white matter hyperintensities), which are both frequent, overlapping and difficult to distinguish [8, 18].

T1 mapping is a quantitative imaging technique being evaluated in multiple neurological diseases to improve diagnostic and predictive measures and to quantify underlying mechanisms even in diseases with only subtle changes [19–21]. These changes can especially be detected in the white matter of the brain and can be related to a variety of white matter microstructural features such as myelin and non-myelin water content, axonal size and axonal density [22, 23]. As the periventricular white matter is involved in primary disease mechanisms in iNPH, aim of this study was to evaluate the diagnostic value of T1 mapping in patients with iNPH and its ability to predict clinical improvement after CSF-TT.

## Methods

### Study Population

In this prospective, single center pilot study T1 mapping of the cerebrum was performed in 15 patients with suspected iNPH prior to and after CSF-TT. T1 mapping was also performed in 8 age-matched, healthy controls without a known history, signs or symptoms of neurological diseases. In addition, controls specifically were only included if they did not show clinical symptoms or imaging characteristics attributable to iNPH on clinical examination or imaging prior to inclusion. Patients were recruited in the Department of Neurology and Neurosurgery of the University Medical Center Göttingen, Germany. T1 mapping was performed at the Max-Planck-Institut für biophysikalische Chemie in Göttingen, Germany as well as at the University Medical Center Göttingen, Germany, on a technically identical magnetic resonance imaging (MRI) device using the same scan protocol.

Inclusion criteria were the presence of gait disturbance as an obligatory symptom of Hakim’s triad as well as a clinical history suspicious for iNPH. Patients included showed gait impairment of varying severity, ranging from a broad-based, unsteady gait pattern with reduction in step height to a “magnetic foot” movement with almost complete incapacity to walk. Cognitive decline and urinary incontinence or urge symptomatic were not mandatory for the inclusion in this study. All iNPH study subjects had received an MRI in a reasonable time window before inclusion showing disproportionate ventricular enlargement. As quantitative criteria for iNPH, Evans index > 0.3 and callosal angle < 90° were considered as pathological [15, 16]. In addition, the extent of white matter hyperintensities has been rated using the Fazekas scale by experienced neuroradiologists [24]. Exclusion criteria were a clinical history of secondary NPH, contraindications for MRI, inability to give informed consent or tolerate supine position for the estimated imaging time.

All patients fulfilling the inclusion criteria received T1 mapping before and within the first 65 h after a CSF-TT (median 22.5 h; IQR 17–24 h). CSF-TT was performed with the patient in an upright position by lumbar puncture (LP) beneath the spinous processes of the 3rd, 4th or 5th lumbar vertebra, and an average volume of 31.3 ml (SD ± 5.3 ml) CSF was removed after measurement of the CSF opening pressure. All patients had cell count, lactate, TAU/phosphoTAU and beta-amyloid 40/42 ratio within the normal range.

The study was carried out in accordance with the code of ethics of the World Medical Association (Declaration of Helsinki) and approved by the ethics committee of the University Medicine Göttingen (29/6/18). All patients and healthy controls gave written informed consent.

### Tests of Gait and Cognition

All patients underwent testing of gait and cognition prior to and after CSF-TT; healthy controls underwent similar consecutive testing at one single timepoint before the MRI. Tests of gait and cognition were performed blinded concerning the results of the conventional MRI and T1 mapping. To quantify gait impairment, we applied the 3 m timed up and go test (TUG test) and the 30m walking test (30MWT). In the TUG test, the patient is asked to rise from a sitting position in a chair, walk 3 m, turn around and sit down again; the needed time for this task is taken as a measure for mobility and interpreted as follows: < 10 s: no gait impairment, 10–19 s: moderate impairment, 20–29 s: functional relevant impairment and > 30 s: severe impairment [25]. In the 30MWT, the number of steps is counted, and the walking time is measured along a distance of 30 m [26]. Both tests have been shown to be effective tools in quantifying gait impairment for diagnosing iNPH [27]. To compare gait measurements prior to and after CSF-TT, improvement in walking time or number of steps is given in percent of the respective data at baseline and later correlated with T1 relaxation times. The improvement of motor and cognitive functions was determined as follows [28]:

$$\textit{Improvement}\,\left(\% \right)=\frac{test\,\textit{result}\,\textit{before}\,TT-test\,\textit{result}\,\textit{after}\,TT}{test\,\textit{result}\,\textit{before}\,TT}*100$$

Improvement of gait measurement tests after CSF-TT (3m TUG in seconds and 30MWT number of steps and seconds) was defined as decrease in time and steps of at least 20% [28, 29]. In cases of a quantitative deterioration of gait parameters zero percent improvement was rated.

Cognitive functions were assessed using two different test: 1) Montreal cognitive assessment test (MoCA) [30] containing a battery of test items, which allows a broad examination of various cognitive functions of memory, attention, perception, verbal fluency and visuospatial functions and 2) the trail making tests A and B [31] representing 2 tests with different levels of difficulty, primarily testing functions of attention, sustained attention, as well as logical thinking, taking into account the time required to perform the test.

Urinary continence was evaluated taking the medical history and by asking the patient and next of kin.

### T1 Mapping and Interpretation of T1 Maps

The MRI studies were conducted at 3 T (Magnetom Prisma, Siemens Healthcare Erlangen, Germany) using a 64-channel head coil. Anatomical images were based on a T2-weighted turbo spin-echo sequence with in-plane resolution of 0.7 mm and a slice thickness of 3 mm in axial sections (repetition time TR = 4280 ms, echo time TE = 89 ms, flip angle 120°, turbo factor: 18, echo train length: 8). Whole brain T1 mapping was performed by acquiring 35 slices.

The T1 mapping technique with an in-plane resolution of 0.5 mm and a slice thickness of 4 mm is based on a single inversion-recovery experiment with a leading slice-selective 180° inversion pulse, a highly undersampled radial gradient-echo readout and a nonlinear inverse image reconstruction technique, for details see [32]. Briefly, the method employs a low-flip angle gradient-echo sequence (TR = 3.81 ms, TE = 2.60 ms, flip angle 6°) with a small golden-angle radial trajectory (angle = 20.89°) and radiofrequency spoiling by random phase alterations [33]. To increase computational speed, binning of the data involved 17 spokes per frame and resulted in a temporal resolution of 65 ms for sampling the inversion-recovery process. The acquisition of a total of 62 images then yielded a measuring time of 4 s per T1 map.

Immediately after completion of data acquisition, maps of T1 relaxation times are automatically calculated and displayed on the MRI system. The values are obtained by a pixelwise fitting of the exponential signal model [34] to the set of reconstructed serial images.

**Fig. 1:**
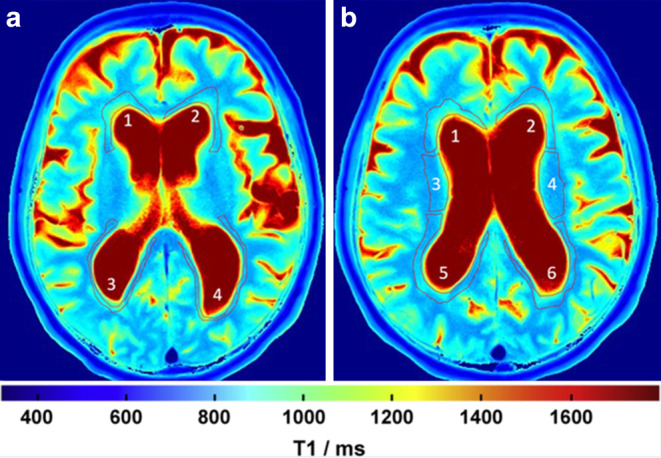
Representative T1 maps of an iNPH case demonstrating the predefined regions of interest (ROIs). **a** Four ROIs manually drawn at the level of the internal capsule (*a1* right inferior anterior horn; *a2* left inferior anterior horn; *a3* right inferior posterior horn; *a4* left inferior posterior horn) and **b** six ROIs drawn at the superior level, on which both lateral ventricles were fully visible (*b1* right superior anterior horn; *b2* left superior anterior horn; *b3* right corona radiata; *b4* left corona radiata; *b5* right superior posterior horn; *b6* left superior posterior horn). The mean T1 relaxation times were analyzed for every ROI

Mean T1 values of the cerebrum were obtained by manually drawing the predefined periventricular regions of interest (ROI) on two axial sections: first was at the level of the internal capsule (four ROIs: A1 right inferior anterior horn; A2 left inferior anterior horn; A3 right inferior posterior horn; A4 left inferior posterior horn) and second at the superior level, on which both lateral ventricles were fully visible (six ROIs: B1 right superior anterior horn; B2 left superior anterior horn; B3 right corona radiata; B4 left corona radiata; B5 right superior posterior horn; B6 left superior posterior horn). A representative case with all ROIs is given in Fig. 1*. *The ROIs were drawn on gray scale T1 maps without artificial color borders and using corresponding T2-weighted anatomical images. To demonstrate inter-rater reliability, a second T1 mapping experienced rater manually determined the predefined ROIs on all baseline T1 maps and the mean T1 relaxation times were compared to the first rater.

**Fig. 2:**
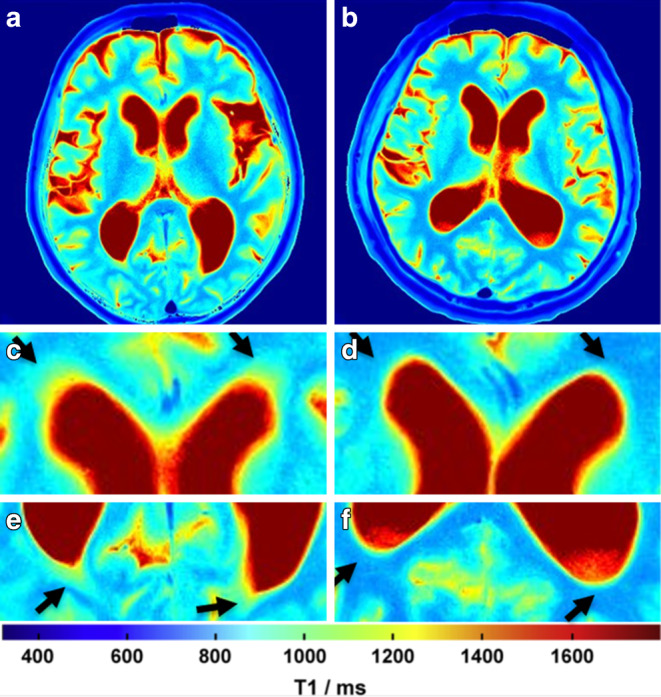
Representative axial T1 maps of a patient fulfilling all clinical criteria for iNPH and with improvement after CSF tap test (CSF-TT; *left column* **a**, **c** and **e**) and a case with an ambiguous clinical presentation without clinical improvement after CSF-TT (*right colums* **b**, **d** and **f**). Note the higher periventricular T1 relaxation times of the patient with high likelihood of iNPH diagnosis (*left column* as well as high T1 relaxation times at the anterior and posterior horn of the lateral ventricle (*black arrows* in **c**, **e**) compared to lower periventricular T1 relaxation times in the patient with unlikely iNPH diagnosis (*right column*) and no increases in T1 relaxation time at the anterior and posterior horn of the lateral ventricle (*black arrows* in **d**, **f**)

Color-coded T1 maps are only used for improved visualization and qualitative analysis (*see* representative cases in Fig. 2 of a typical iNPH T1 map and a T1 map of a patient in whom the diagnosis of iNPH was unlikely). Data analysis and ROI definition were performed using Fiji, an open-source image processing package based on ImageJ [35].

### Statistical Analysis

Statistical analysis was performed using GraphPad Prism 6.0 (GraphPad Software, San Diego, CA, USA). Baseline characteristics of all patients and controls are shown as mean ± standard deviation (SD), if normally distributed, and as median with interquartile range (IQR), if not. T1 relaxation times were compared using Student’s *t*-test. Due to recommendations for studies of an exploratory nature no Bonferroni adjustments for *p*-values were performed. Correlations between clinical or radiological scores and T1 relaxation times were determined by a bivariate Pearson correlation if normally distributed. Otherwise, a Spearman’s rank correlation was applied. *P*-values below 0.05 were considered as statistically significant. To demonstrate inter-rater reliability of T1 relaxation time measurements, the intraclass correlation coefficient was used.

## Results

### Patient Characteristics

Of the 15 patients with iNPH, mean age was 77.8 ± 6.5 years (controls: 75.6 ± 12.2 years, *p* = 0.580) and 12 (80%) patients were male (1 male control, 20%) (Table 1). Gait disturbances were found in all 15 iNPH patients, 13 (86.7%) had additional cognitive impairment (MoCA < 26 points) and urinary incontinence or urge was found in 8 (53.3%) of iNPH patients. In the control group, one (12.5%) patient had cognitive impairment; gait disturbances and urinary incontinence were not found in the control group. The iNPH patients performed significantly worse on motor and cognitive function at baseline with most pronounced differences in the 3m-TUG (16.5 s, IQR 11.8–36 s vs. 7.3 s, IQR 6.4–9.4 s, *p* = 0.002) and 30MWT (37.6 s, IQR 28.1–57.5s vs. 20.5 s, IQR 17.8–23.7 s, *p* = 0.002).

**Table 1:** Baseline characteristics of patients with idiopathic normal pressure hydrocephalus and healthy controls

	iNPH (*n* = 15)	Controls (*n* = 8)	*p*-value
Age (years, mean ± SD)	77.8 ± 6.5	75.6 ± 12.2	0.580
Sex male (*n*, %)	12 (80)	1 (20)	–
Gait disturbance (*n*, %)	15 (100)	0 (0)	–
Cognitive Impairment^a^ (*n*, %)	13 (86.7)	1 (20)	–
Urinary incontinence or urge (*n*, %)	8 (53.3)	0 (0)	–
Hakims triad fulfilled (*n*, %)	7 (46.7)	0 (0)	–
CSF pressure (mean cmH2O ± SD)	18.4 ± 4.6	n.a.	–
CSF removal (mean ml ± SD)	31.3 ± 5.3	n.a.	–
Time between CSF-TT and 2nd MRI (median hours, IQR)	22.5 (17–24)	n.a.	–
Evans index (mean ± SD)	0.37 ± 0.03	0.28 ± 0.04	0.002
Callosal angle (mean ± SD)	86.9 ± 24	142.3 ± 16.6	0.003
Fazekas scale (median, IQR)	1 (0–2.5)	1 (0–1)	0.497
Shunt indication after spinal tap test (*n*, %)	7 (46.6)	n.a.	–
Clinical tests at baseline			
3m TUG test (median s, IQR)	16.5 (11.8–36)	7.3 (6.4–9.4)	0.002
30MWT (median steps, IQR)	60 (48–68.5)	41 (37–46.5)	0.028
30MWT (median s, IQR)	37.6 (28.1–57.5)	20.5 (17.8–23.7)	0.002
MoCA (median score, IQR)	22 (19.5–24)	28 (22–28.5)	0.065
Trail making test A (median s, IQR)	63.8 (46.5–91.3)	38.1 (24–56.6)	0.046
Trail making test B (median s, IQR)	193.5 (123.9–282.3)	94.2 (51–181.8)	0.078

*iNPH* idiopathic normal pressure hydrocephalus, *SD* standard deviation, *CSF* cerebrospinal fluid, *CSF-TT* CSF-tap test, *MRI* magnetic resonance imaging, *IQR* interquartile range, *TUG* timed-up-and-go test, *30MWT* 30m walking test, *MoCA* Montreal cognitive assessment, *n.a.* not applicable

^a^cognitive impairment was defined as Montreal cognitive assessment score < 26 points

On MRI, all iNPH patients showed ventricular enlargement and the Evans index (0.37 ± 0.03 vs. 0.28 ± 0.04, *p* = 0.002) was larger as well as the callosal angle (86.9° ± 24° vs. 142.3° ± 16.6°, *p* = 0.003) lower in the iNPH group. White matter hyperintensities, quantified by the Fazekas scale, were comparable between groups (Fazekas scale 1 (0–2.5) vs. 1 (0–1), *p* = 0.497). CSF pressure in the iNPH group was in the normal range (18.4 ± 4.6 cmH_2_O) and the mean amount of removed CSF was 31.3 ± 5.3 ml. After CSF-TT, 12 (80%) iNPH patients showed clinical improvement and in 7 (46.6%) iNPH patients permanent shunting was indicated.

### T1 Mapping

**Fig. 3:**
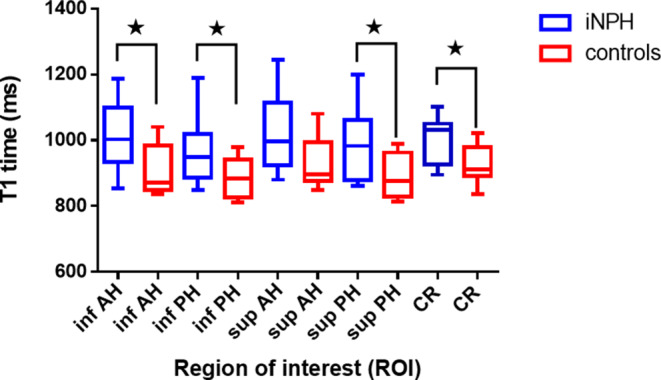
Mean T1 relaxation times in patients with idiopathic normal pressure hydrocephalus (*iNPH*; *blue boxplots*) and controls (*red boxplots*) in predefined, periventricular regions of interest (ROIs) prior to CSF tap test. *Inf* inferior, *AH* anterior horn of the lateral ventricle, *PH* posterior horn, *sup* superior, *CR* corona radiata; *indicates a *p*-value < 0.05 (see Table 2)

Representative cases demonstrating the ROI selection and qualitative differences in color-coded T1 maps are shown in Figs. 1 and 2. On baseline scans, T1 relaxation times in all ROIs were higher in iNPH patients compared to controls with highest differences found in the inferior anterior horn (1006.1 ± 93.1 ms vs. 911.4 ± 77.4 ms; *p* = 0.023, ∆T1 = 94.7 ms) and the superior posterior horn (982.9 ± 103.1 ms vs. 892.5 ± 68 ms; *p* = 0.037, ∆T1 = 90.4 ms) of the lateral ventricles (see Table 2 and Fig. 3).

**Table 2:** Baseline mean T1 relaxation times prior to CSF-tap test in patients with idiopathic normal pressure hydrocephalus (iNPH) and healthy controls

	T1 (ms)		*p*-value
	iNPH	Controls	
Inferior anterior horn (mean left and right ± SD)	1006.1 ± 93.1	911.4 ± 77.4	0.023
Inferior posterior horn (mean left and right ± SD)	968.6 ± 97.7	884.1 ± 60.5	0.038
Superior anterior horn (mean left and right ± SD)	1022.7 ± 114.3	933 ± 81	0.063
Superior posterior horn (mean left and right ± SD)	982.9 ± 103.1	892.5 ± 68	0.037
Corona radiata (mean left and right ± SD)	1001.8 ± 72.5	924.4 ± 59.4	0.017

*iNPH* idiopathic normal pressure hydrocephalus, *SD* standard deviation, *CSF* cerebrospinal fluid

Inter-rater agreement was excellent for all ROIs (intraclass correlation coefficient > 0.94, *p* < 0.001; *see *supplementary table 1). In a next step, T1 relaxation times prior to CSF-TT were compared to T1 relaxation times after the CSF-TT. As shown in supplementary table 2 and supplementary figure 1, mean T1 relaxation times in the inferior anterior horn (∆ T1 = −29.6 ms), the inferior posterior horn (∆ T1 = −26.2 ms) and superior posterior horn (∆ T1 = −16.2 ms) dropped from the pre-CSF-TT scan to the post-CSF-TT scan. These drops were statistically not significant (*p* > 0.4) and there was no drop of the mean T1 relaxation time in all other regions (superior anterior horn mean ∆ T1 = 2 ms; corona radiata mean ∆ T1 = 0 ms).

### T1 Relaxation Times, Conventional MRI, Cognitive and Motor Tests

As shown in supplementary table 3, we found a significant correlation between higher T1 relaxation times in all ROIs and higher age as well as a positive correlation between higher T1 relaxation times and higher Fazekas scale around the inferior anterior horn (r = 0.725, *p* = 0.001), around the superior anterior horn (r = 0.594, *p* = 0.013) and in the corona radiata (*p* = 0.572, *p* = 0.018) on baseline imaging. There was a consistent negative correlation between periventricular T1 relaxation times and the callosal angle as well as a consistent positive correlation between the T1 relaxation times and the Evans index, both reaching no statistical significance.

Worse baseline cognitive test performance was associated with higher periventricular T1 relaxation time. High T1 relaxation times in all ROIs were negatively correlated with low MoCA scores and there was a significant positive correlation between longer times to perform the trail making test A and T1 relaxation times in the inferior anterior horn (r = 0.657, *p* = 0.007) and the superior anterior horn (r = 0.629, *p* = 0.011) (see Table 3).

**Table 3:** Correlation between T1 relaxation times and baseline cognitive tests in patients with idiopathic normal pressure hydrocephalus and controls

	MoCA score		Trail making test A (s)		Trail making test B (s)	
	r	*p*-value	r	*p*-value	r	*p*-value
T1 (ms) inferior anterior horn (mean left and right)	−0.682	0.001	0.657	0.007	0.245	0.359
T1 (ms) inferior posterior horn (mean left and right)	−0.535	0.018	0.395	0.131	0.180	0.504
T1 (ms) superior anterior horn (mean left and right)	−0.613	0.005	0.629	0.011	0.274	0.303
T1 (ms) superior posterior horn (mean left and right)	−0.526	0.021	0.458	0.076	0.251	0.348
T1 (ms) corona radiata (mean left and right)	−0.587	0.008	0.439	0.090	0.121	0.655

*MoCA* Montreal cognitive assessment

Quantitative gait measurements at baseline showed significant correlations with periventricular T1 relaxation times (see Table 4). Longer times to perform the 3m-TUG and the 30MWT at baseline were positively correlated with higher periventricular T1 relaxation times with strongest correlations for periventricular inferior anterior and superior anterior horn regions. The iNPH patients with improvement of > 20% in the 3m-TUG had higher periventricular T1 relaxation times with highest differences compared to non-improvement iNPH patients for the periventricular regions around the inferior anterior horn (1079 ± 93 ms vs. 982 ± 83 ms; *p* = 0.087) and the inferior posterior horn (1048 ± 111 ms vs. 922 ± 66 ms; *p* = 0.032). Higher improvement of the time to perform the 3m-TUG showed higher periventricular T1 relaxation times at baseline. These positive correlations were statistically significant for the periventricular inferior anterior horn (r = 0.609, *p* = 0.027), inferior posterior horn (r = 0.677, *p* = 0.011) and superior anterior horn regions (r = 0.630, *p* = 0.021).

**Table 4:** Correlation between T1 relaxation times and gait function in all study subjects (patients with idiopathic normal pressure hydrocephalus, iNPH and controls) for baseline testing (*n* = 23) and for the iNPH group for improvement (*n* = 15)

	3 m-TUG (s)		Impr. in TUG test (%)		30MWT (steps)		Impr. in 30 m steps (%)		30MWT (s)		Impr. in 30 m time (%)	
	r	*p*-value	r	*p*-value	r	*p*-value	r	*p*-value	r	*p*-value	r	*p*-value
T1 (ms) inferior anterior horn (mean left and right)	0.571	0.019	0.609	0.027	0.365	0.150	−0.155	0.631	0.754	0.001	0.531	0.062
T1 (ms) inferior posterior horn (mean left and right)	0.402	0.111	0.677	0.011	0.319	0.212	−0.303	0.338	0.671	0.004	0.488	0.091
T1 (ms) superior anterior horn (mean left and right)	0.574	0.018	0.630	0.021	0.407	0.106	−0.052	0.873	0.787	< 0.001	0.550	0.052
T1 (ms) superior posterior horn (mean left and right)	0.409	0.104	0.511	0.074	0.304	0.235	−0.240	0.453	0.661	0.005	0.461	0.113
T1 (ms) corona radiata (mean left and right)	0.395	0.118	0.288	0.34	0.270	0.294	−0.265	0.405	0.614	0.010	0.412	0.164

*3* *m‑TUG* 3m timed up and go test, *30MWT* 30‑m walking test, *Impr.* improvement

## Discussion

In this exploratory study, we for the first time investigated the diagnostic and predictive potential of quantitative T1 mapping in patients with iNPH. In our study, periventricular T1 relaxation times were significantly increased in patients with iNPH and high T1 values in these regions were correlated with improvement of gait in this patient group.

Periventricular white matter hyperintensities are a frequent finding in patients with iNPH as well as in patients with small vessel disease, in whom they tend to be more likely to be disseminated in the deep white matter [36]; however, a typical hallmark of iNPH is white matter changes located around the anterior and posterior top of the lateral ventricles, likely to be caused by CSF egress into the white matter resulting from pulsatile ICP elevations [1, 17]. This egress causes disruption of the ependyma, edema, neuronal degeneration as well as gliosis [37] and often occurs in combination with diffusely distributed white matter hyperintensities associated with vascular risk factors, also characterized by demyelination and axon loss [38]. Both kinds of white matter changes seem to represent pathomorphological changes and likely irreversible disease activity. In this respect, several studies showed worse surgical outcome in cases of increased white matter lesions [3, 12, 39]. Nonetheless, deep white matter hyperintensities in iNPH patients should not be used as an exclusion criterion when indicating VP shunt implantation [36, 40].

Deep white matter hyperintensities were present to a comparable extent (Fazekas scale) in iNPH patients and controls in our study. Most interestingly, we found significant differences in T1 relaxation times at the superior anterior and inferior posterior horns of the lateral ventricle. In these regions CSF translocation is most frequently seen in iNPH patients. Therefore, T1 mapping represents an imaging technique not only visualizing subtle changes in this region, but also quantifying these changes. T1 relaxation times, however, seem to increase both with higher concentrations of CSF in the white matter (higher water content caused by CSF ependymal translocation) as well as with demyelination and axon loss (caused by small vessel disease), which both have been demonstrated in other studies [19, 22, 41, 42]. Therefore, T1 mapping might help to enrich the diagnostic work-up of iNPH to better distinguish imaging and clinical characteristics from patients with predominant small vessel disease, which normally do not improve after CSF-TT. In this respect, however, more scans in iNPH and age-matched controls are necessary to define cut-off values for periventricular T1 relaxation times that discriminate iNPH from other diseases like small vessel encephalopathy or AD.

Patients with iNPH show clinical improvement (especially in gait motor function) after CSF-TT [3]. In the iNPH group, this improvement was seen in 80% of cases, which is in the upper range of reported response rates of other studies [3, 29] and might indicate a selection bias in our study. On the contrary, this high response rate more likely might reflect the fact that the CSF-TT procedure and its interpretation vary widely between studies and centers [3]. Previous studies also reported significantly slower gait caused by shorter steps, higher stride variation and lower cadence [3, 29]. We quantified these typical gait changes in iNPH patients with the 3m-TUG and the 30MWT. We showed a significant correlation between periventricular T1 relaxation times and the time of the 3m-TUG (and, with a lesser degree of correlation the time of the 30MWT), indicating that the T1 relaxation times could be useful as a marker for disease severity. As the 3m-TUG also tests elements such as balance and coordination, these functions could more likely be associated with increased periventricular T1 relaxation times when compared to walking distance and walking speed. On the other hand, correlations in small sample sizes (like in our study) must be interpreted with caution as very small changes in measurements can have large impacts on correlation coefficients. Given the exploratory character of our study, the interpretation of the results therefore must be handled with caution. As already mentioned, also this application of T1 mapping requires far larger patient groups, especially to determine age and small vessel hyperintensity corrected cut-off values.

Although 80% of iNPH patients in our study responded to the CSF-TT, no significant reductions in the periventricular T1 relaxation times were found after the tapping procedure. This finding likely reflects the multifactorial etiology of the disease, as only a part of the white matter hyperintensities in iNPH patients are potentially reversible. In this respect, a study by Kamyia et al. showed reversible and irreversible changes along the corticospinal tract in iNPH patients using diffusion tensor imaging [43]. As in almost all periventricular ROIs the mean T1 relaxation times in the follow-up scans after CSF-TT tended to be lower, it can be speculated that also small changes in T1 relaxation times are correlated with clinical improvement and that the reduction in T1 relaxation times may be caused by a reduction in CSF content in the periventricular white matter. On the other hand, given the high specificity of T1 mapping to detect microstructural white matter changes, it can be speculated that T1 relaxation times are either altered early after the CSF-TT or late after the CSF-TT. Clinical improvements are normally seen within 24 h after CSF-TT [44] (within the time frame of the follow-up scans in our study), and therefore the lack of sufficient decrease in T1 relaxation times is unexpected and might indicate a lack of correlation of imaging and clinical parameters after CSF-TT. In addition, although the T1 relaxation times were significantly correlated with cognitive function, we did not perform an analysis on whether cognitive functions are improved after CSF-TT, as within a 24 h time frame a significant learning effect can be assumed.

A strength of this study is a thorough patient selection and the application of a novel imaging technique in iNPH patients with a high sensitivity to detect white matter changes. Limitations are inherent to the exploratory character of this study with a small number of patients and controls as well as a lack of long-term follow-ups. In addition, our data only cover the effects of a single CSF-TT and are not able to provide insights concerning transient lumbar or permanent ventriculoperitoneal shunting. In addition, deep white matter hyperintensities and age are major contributing factors to increased periventricular T1 relaxation times [45, 46]. Although age and extent of white matter hyperintensities were comparable between iNPH patients and controls, this finding is still able to confound the correlation between T1 relaxation times and gait measurements at baseline; however, the influence of age and white matter hyperintensities does not explain the correlation of T1 relaxation times and improvement of gait measurements, which implies a specific relationship between increase of T1 relaxation times and responsiveness to the CSF-TT.

In conclusion, our exploratory study provides preliminary evidence that increased periventricular T1 relaxation times may be useful (i) to differentiate iNPH patients from other frequent differential diagnoses and (ii) to predict clinical response to CSF-TT. Larger studies with long-term follow-ups after permanent shunting are needed to show long-term effects of CSF drainage on periventricular T1 relaxation times and to determine reliable cut-off T1 values for the differential diagnosis and treatment response in this patient group.

## Supplementary Information

**Supplementary fig 1: **Change in mean T1 relaxation times prior (*dark blue boxplots*) and after (*light blue boxplots*) CSF tap test. The drops of the T1 relaxation times in the inferior anterior horn, the inferior posterior horn and superior posterior horn were statistically not significant (*p* > 0.4). *Inf* inferior; *AH* anterior horn of the lateral ventricle, *PH* posterior horn, *sup* superior; *CR* corona radiata; * indicates a *p*-value < 0.05 (see Table 2).

Supplementary table 1: Inter-rater reliability of T1 relaxation time measurements

Supplementary table 2: T1 relaxation times prior to and after CSF tap test in patients with idiopathic normal pressure hydrocephalus (iNPH)

Supplementary table 3: Correlations of age and conventional imaging parameters with periventricular T1 relaxation times in patients with idiopathic normal pressure hydrocephalus and controls

## Abbreviations

30MWT: 30‑m walking test
CSF: Cerebrospinal fluid
CSF-TT: Cerebrospinal fluid tap test
iNPH: Idiopathic normal pressure hydrocephalus
IQR: Interquartile range
MoCA: Montreal cognitive assessment
ROI: Region of interest
SD: Standard deviation
TUG test: 3- m timed up and go test
