# Supplementary figures and images for: High Periventricular T1 Relaxation Times Predict Gait Improvement After Spinal Tap in Patients with Idiopathic Normal Pressure Hydrocephalus
Source: Clin Neuroradiol. 2022 Apr 7;32(4):1067–76. doi: 10.1007/s00062-022-01155-0 (PMC9744711; doi:10.1007/s00062-022-01155-0)

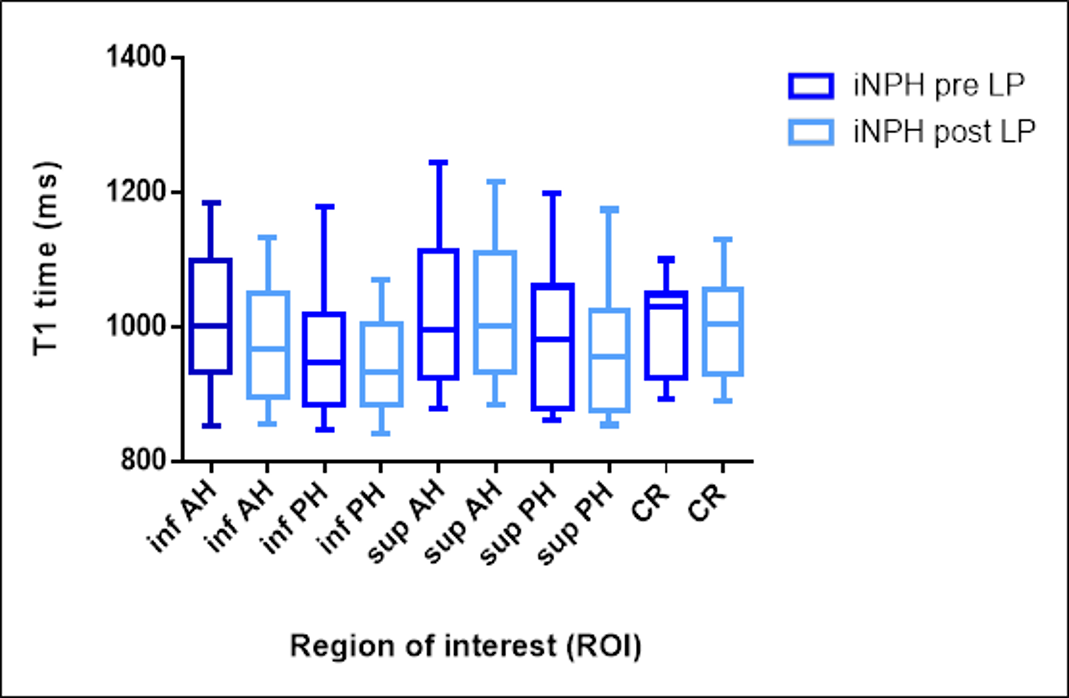

Supplement: Supplementary file 1 — Supplementary fig 1: Change in mean T1 relaxation times prior (dark blue boxplots) and after (light blue boxplots) CSF tap test. The drops of the T1 relaxation times in the inferior anterior horn, the inferior posterior horn and superior posterior horn were statistically not significant (p > 0.4). Inf inferior; AH anterior horn of the lateral ventricle, PH posterior horn, sup superior; CR corona radiata; * indicates a p-value < 0.05 (see Table 2). [file 62_2022_1155_MOESM1_ESM.tiff]
