# Supplementary material for: High Periventricular T1 Relaxation Times Predict Gait Improvement After Spinal Tap in Patients with Idiopathic Normal Pressure Hydrocephalus
Source: Clin Neuroradiol. 2022 Apr 7;32(4):1067–76. doi: 10.1007/s00062-022-01155-0 (PMC9744711; doi:10.1007/s00062-022-01155-0)
Supplement: Supplementary file 2 — Supplementary table 1: Inter-rater reliability of T1 relaxation time measurements [file 62_2022_1155_MOESM2_ESM.docx]

|  |  |  |
| --- | --- | --- |
| **Supplementary table 1:** Inter rater reliability of T1-relaxation time measurements | | |
|  |  |  |
|  | T1 (ms) | |
|  | ICC (95% CI) | p-value |
| Inferior anterior horn | 0.981 (0.938-0.995) | <0.001 |
| Inferior posterior horn | 0.954 (0.799-0.988) | <0.001 |
| Superior anterior horn | 0.949 (0.830-0.985) | <0.001 |
| Superior posterior horn | 0.979 (0.929-0.994) | <0.001 |
| Corona radiata | 0.993 (0.974-0.998) | <0.001 |
| ICC: intra class correlation coefficient | | |
|  |  |  |
