# Supplementary material for: High Periventricular T1 Relaxation Times Predict Gait Improvement After Spinal Tap in Patients with Idiopathic Normal Pressure Hydrocephalus
Source: Clin Neuroradiol. 2022 Apr 7;32(4):1067–76. doi: 10.1007/s00062-022-01155-0 (PMC9744711; doi:10.1007/s00062-022-01155-0)
Supplement: Supplementary file 3 — Supplementary table 2: T1 relaxation times prior to and after CSF tap test in patients with idiopathic normal pressure hydrocephalus (iNPH) [file 62_2022_1155_MOESM3_ESM.docx]

|  |  |  |  |  |
| --- | --- | --- | --- | --- |
| **Supplementary table 2:** T1 relaxation times prior to and after CSF- tap test in patients with idiopathic normal pressure hydrocephalus (iNPH) | | | | |
|  |  |  |  |  |
|  | T1 (ms) | | Δ T1 (ms) | p-value |
|  | iNPH pre CSF-TT | iNPH post CSF-TT |  |  |
| Inferior anterior horn (mean left and right ± SD) | 1006 ± 93.2 | 976.4 ± 91.3 | .-29.6 | 0.409 |
| Inferior posterior horn (mean left and right ± SD) | 967.7 ± 96 | 941.5 ± 71 | .-26.2 | 0.426 |
| Superior anterior horn (mean left and right ± SD) | 1023 ± 114.3 | 1025 ± 109.2 | 2 | 0.947 |
| Superior posterior horn (mean left and right ± SD) | 982.8 ± 103.1 | 966.6 ± 93.6 | .-16.2 | 0.663 |
| Corona radiata (mean left and right ± SD) | 1002 ± 72.6 | 1002 ± 74.9 | 0 | 0.999 |
| CSF-TT: cerebrospinal fluid tap test; SD: standard deviation | | |  |  |
|  |  |  |  |  |
