# Supplementary material for: High Periventricular T1 Relaxation Times Predict Gait Improvement After Spinal Tap in Patients with Idiopathic Normal Pressure Hydrocephalus
Source: Clin Neuroradiol. 2022 Apr 7;32(4):1067–76. doi: 10.1007/s00062-022-01155-0 (PMC9744711; doi:10.1007/s00062-022-01155-0)
Supplement: Supplementary file 4 — Supplementary table 3: Correlations of age and conventional imaging parameters with periventricular T1 relaxation times in patients with idiopathic normal pressure hydrocephalus and controls [file 62_2022_1155_MOESM4_ESM.docx]

|  |  |  |  |  |  |  |  |  |
| --- | --- | --- | --- | --- | --- | --- | --- | --- |
| **Supplementary table 3:** Correlations of age and conventional imaging parameters with periventricular T1 relaxation times in patients with idiopathic normal pressure hydrocephalus and controls | | | | | | | | |
|  |  |  |  |  |  |  |  |  |
|  | Age (years) | | Callosal Angel (°) | | Evans Index | | Fazekas score | |
|  | r | p-value | r | p-value | r | p-value | r | p-value |
| T1 (ms) inferior anterior horn (mean left and right) | 0.485 | 0.019 | -0.312 | 0.258 | 0.270 | 0.294 | 0.725 | 0.001 |
| T1 (ms) inferior posterior horn (mean left and right) | 0.383 | 0.071 | -0.151 | 0.592 | 0.076 | 0.771 | 0.410 | 0.104 |
| T1 (ms) superior anterior horn (mean left and right) | 0.468 | 0.024 | -0.136 | 0.629 | 0.096 | 0.714 | 0.594 | 0.013 |
| T1 (ms) superior posterior horn (mean left and right) | 0.492 | 0.017 | -0.335 | 0.223 | 0.138 | 0.596 | 0.480 | 0.053 |
| T1 (ms) corona radiata (mean left and right) | 0.458 | 0.028 | -0.274 | 0.323 | 0.176 | 0.499 | 0.572 | 0.018 |
|  |  |  |  |  |  |  |  |  |
